# Supplementary material for: Divergent roles of RIPK3 and MLKL in high-fat diet–induced obesity and MAFLD in mice
Source: Life Sci Alliance. 2024 Nov 12;8(1):e202302446. doi: 10.26508/lsa.202302446 (PMC11557689; doi:10.26508/lsa.202302446)
Supplement: Supplementary file 8 [file LSA-2023-02446_TableS1.docx]

**Table S1. qPCR primers**

| **Gene** | **Forward primer 5’ – 3’** | **Reverse Primer 5’ – 3’** |
| --- | --- | --- |
| *Abcd2* | CCAACGGTTGTGGGAAAAGC | GAGACATGTATGGCCTCTGTGG |
| *Acaca* | GATGAACCATCTCCGTTGGC | GACCCAATTATGAATCGGGAGTG |
| *Acca1b* | CAGGACGTGAAGCTAAAGCCT | CTCCGAAGTTATCCCCATAGGAA |
| *Acot3* | TGCTCAGTCACCCTCAGGTAA | GCTTGGTGTTTATGCCCACG |
| *Cd36* | TGTGTTTGGAGGCATTCTCA | TTTTGCACGTCAAAGATCCA |
| *Cidea* | GTGGTGGACACAGAGGAGTTC | TGGGACATACTTACTACCCGGTG |
| *Dgat1* | TGGTAGTGGGCCCAAGGTAG | TGCAGACGATGGCACCTCAG |
| *Elov6* | GAAAAGCAGTTCAACGAGAACG | AGATGCCGACCACCAAAGATA |
| *Fabp4* | AAGGTGAAGAGCATCATAACCCT | TCACGCCTTTCATAACACATTCC |
| *Fasn* | CTGACTCGGCTACTGACACG | AATGGGGTGCACAAGGAACA |
| *Casp8* | CTCCGAAAAATGAAGGACAGA | CGTGGGATAGGATACAGCAGA |
| *Ccl2* | GTTGGCTCAGCCAGATGCA | AGCCTACTCATTGGGATCATCTTG |
| *Cxcl1* | CCTTGACCCTGAAGCTCCCT | CAGGTGCCATCAGAGCAGTCT |
| *Hmgcr* | CGTAAGCGCAGTTCCTTCC | TTGTAGCCTCACAGTCCTTGG |
| *Hmgcs2* | GAGGGCATAGATACCACCAACG | AATGTCACCACAGACCACCAGG |
| *Il1b* | AGTTGACGGACCCCAAAAG | AGCTGGATGCTCTCATCAGG |
| *Ldlr* | AGGCTGTGGGCTCCATAGG | TGCGGTCCAGGGTCATCT |
| *Mlkl* | TCGATTCTCCCAACATCTTG | GGTGTAGCCTGTATAAGCCTCTG |
| *Mogat1* | TTGTCCTCGGAGGTGCAAAG | CTGGAACCAAACTGGCACCAT |
| *Nlrp3* | attacccgcccgagaaagg | tcgcagcaaagatccacacag |
| *Plin4* | TCAGTGGAGGAGTGTGGTCA | ACTGCCAGCTGAGCTTGTTC |
| *Ppara* | AGAGCCCCATCTGTCCTCTC | ACTGGTAGTCTGCAAAACCAAA |
| *Pparg* | GGAAGACCACTCGCATTCCTT | GTAATCAGCAACCATTGGGTCA |
| *Scd1* | CACCTGCCTCTTCGGGATTT | GGCCCATTCGTACACGTCAT |
| *Srebp1* | AACTTTTCCTTAACGTGGGCCT | ATGAGCTGGAGCATGTCTTCG |
| *Srebp2* | GCGCCAGGAGAACATGGT | CGATGCCCTTCAGGAGCTT |
| *Tnf* | CCACCACGCTCTTCTGTCTA | CACTTGGTGGTTTGCTACGA |
| *Vldlr* | GAGGTCAACTGCCCTTCTCG | AGCCATCAACACAGTCTCGG |
| *18s* | GTAACCCGTTGAACCCCATT | CCATCCAATCGGTAGTAGCG |
